# Reliability of Trachoma Clinical Grading—Assessing Grading of Marginal Cases

**DOI:** 10.1371/journal.pntd.0002840

**Published:** 2014-05-01

**Authors:** Salman A. Rahman, Sun N. Yu, Abdou Amza, Sintayehu Gebreselassie, Boubacar Kadri, Nassirou Baido, Nicole E. Stoller, Joseph P. Sheehan, Travis C. Porco, Bruce D. Gaynor, Jeremy D. Keenan, Thomas M. Lietman

**Affiliations:** 1 F.I. Proctor Foundation, San Francisco, California, United States of America; 2 Programme Nationale des Soins Oculaire, Niamey, Niger; 3 The Carter Center Ethiopia, Addis Ababa, Ethiopia; 4 Department of Ophthalmology, University of California, San Francisco, San Francisco, California, United States of America; 5 Department of Epidemiology & Biostatistics, University of California, San Francisco, San Francisco, California, United States of America; University of California San Diego School of Medicine, United States of America

## Abstract

**Background:**

Clinical examination of trachoma is used to justify intervention in trachoma-endemic regions. Currently, field graders are certified by determining their concordance with experienced graders using the kappa statistic. Unfortunately, trachoma grading can be highly variable and there are cases where even expert graders disagree (borderline/marginal cases). Prior work has shown that inclusion of borderline cases tends to reduce apparent agreement, as measured by kappa. Here, we confirm those results and assess performance of trainees on these borderline cases by calculating their reliability error, a measure derived from the decomposition of the Brier score.

**Methods and Findings:**

We trained 18 field graders using 200 conjunctival photographs from a community-randomized trial in Niger and assessed inter-grader agreement using kappa as well as reliability error. Three experienced graders scored each case for the presence or absence of trachomatous inflammation - follicular (TF) and trachomatous inflammation - intense (TI). A consensus grade for each case was defined as the one given by a majority of experienced graders. We classified cases into a unanimous subset if all 3 experienced graders gave the same grade. For both TF and TI grades, the mean kappa for trainees was higher on the unanimous subset; inclusion of borderline cases reduced apparent agreement by 15.7% for TF and 12.4% for TI. When we assessed the breakdown of the reliability error, we found that our trainees tended to over-call TF grades and under-call TI grades, especially in borderline cases.

**Conclusions:**

The kappa statistic is widely used for certifying trachoma field graders. Exclusion of borderline cases, which even experienced graders disagree on, increases apparent agreement with the kappa statistic. Graders may agree less when exposed to the full spectrum of disease. Reliability error allows for the assessment of these borderline cases and can be used to refine an individual trainee's grading.

## Author Summary

Trachoma is the leading infectious cause of blindness and the World Health Organization plans to eliminate it as a public health concern worldwide by the year 2020. This effort in large part involves mass oral antibiotic distributions to communities. A simplified trachoma grading scale is used to assess presence of active infection. Field workers must be properly trained and certified to perform these eye exams because their findings inform when to start and stop community-wide antibiotic treatments. Certification involves measuring agreement in trachoma grades between a trainee and an experienced grader on a test-set of trachoma photographs. Often, these test-sets have hard-to-grade cases of trachoma removed. We found that removing these borderline cases inflates agreement. Including these borderline cases in the test-set allows a more realistic estimate of agreement, but it is still difficult to assess a trainee's grades for cases which even experts disagree on. We found that reliability error, a measure derived from the decomposition of the Brier score (the mean squared error of a set of forecasts), can be used to assess a trainee's evaluation of these borderline cases.

## Introduction

The World Health Organization (WHO) recommends clinical examination of the upper tarsal conjunctiva of children for trachoma to determine when to start and stop mass antibiotic distributions, and when to declare elimination as a public health concern [1]–[4]. A considerable portion of the evidence justifying interventions is based on the clinical examination as primary or secondary outcomes [5]–[10]. Laboratory diagnostic tests for *Chlamydia trachomatis*, the causative agent of trachoma, are relatively expensive and rarely performed in trachoma-endemic areas, so the clinical examination will likely remain important in the future [11].

Clinical grades are assigned using the WHO's simplified grading system, which has 2 grading classes instead of 4, as compared to its predecessor. The simplified grading system was developed for use by trained non-specialist personnel to obtain reliable information on trachoma in population-based surveys or for the simple assessment of the disease at the community level. Trachoma programs almost universally use the simplified system. While its predecessor is able to more finely discern disease activity, it requires more training to use accurately [12].

Agreement with experienced trachoma graders using a kappa statistic is the most common method currently used for certifying competence of field graders [13]–[15]. Unfortunately, clinical trachoma grading can be extremely variable. Even experienced graders disagree on the marginal cases [11]. It could be argued that little information is gained from these marginal cases; if 50% of experienced graders declare a case clinically active, then a trainee's evaluation, whether positive or negative, reveals little. Statistics such as *reliability error*, a measure derived from the decomposition of the Brier score, can assess grading in these marginal cases.

The Brier Score is the mean squared error of a set of predictions. It can be decomposed into three terms: reliability error, resolution, and uncertainty. Reliability error measures how often a set of predictions given the same forecast probability came true. Resolution measures whether different classifications of forecasts in fact had different outcomes, and uncertainty measures the variance of the outcomes, having nothing to do with the forecasts themselves. Decompositions of the Brier score have been used in meteorology to assess accuracy of weather forecasts [16], [17]. Here, we assessed trachoma grading agreement using photographs from a trachoma-endemic area of Niger, estimating inter-grader agreement using both the kappa statistic and reliability error.

## Methods

The Partnership for the Rapid Elimination of Trachoma (PRET) was a three-country community-randomized trial (clinicaltrials.gov trial NCT00792922) which evaluated different mass antibiotic treatment regimens for trachoma [18]. The Niger study site was located in the Matameye district of the Zinder region in Niger. Government health units were chosen from six health centers (Centres de Santé Intégrée [CSI]) and are referred to as communities in this manuscript. Included in the PRET study were 48 communities with 250–600 inhabitants and ≥10% prevalence of active trachoma (trachomatous inflammation - follicular [TF] and/or trachomatous inflammation – intense [TI] per the WHO's simplified trachoma grading system) in children 0–72 months of age [2].

During the PRET baseline visit, in Spring 2010, three trained photographers took two or more photographs of the upper right everted conjunctiva of each study participant in the 48 communities with a Nikon D-series camera with a Micro Nikkor 105 mm f/2.8 lens (Nikon, Tokyo, Japan). Of the 48 communities in PRET, 6 met inclusion criteria for this study, having a prevalence of TF between 40% and 60% among children aged 0–9. The mean pre-treatment TF prevalence in these communities was 51.4%. In total, approximately 1800 photographs were taken of 590 children from these 6 communities. Of those photographs, 200 (11%) were selected for inclusion in this study because they were well focused, centered, and without excessive tears. Specifically, photos were not chosen based on clinical activity, so they presumably represented the entire spectrum of disease including borderline WHO grades.

The 200 photos were compiled into an examination to certify potential trachoma graders. We trained 18 potential graders in the WHO simplified grading system for a trachoma study in Ethiopia, and all 18 took the certification examination. Trainees varied in their prior field experience. 4 trainees were novices, 4 had participated in 1 study-visit, 1 had participated in 2 study-visits, 3 had participated in 3 study-visits, 1 had participated in 4 study-visits, and 5 trainees had participated in 6+ study-visits.

### Analysis

In addition to the 18 trainees, three experienced graders (TML, BDG, JDK) graded each of the 200 cases as either having TF or no TF and as having TI or no TI. Each was masked to the others' grades. A consensus grade was defined for each case as the one that at least 2 of the 3 experienced graders agreed upon. Cases for which all three experienced graders were in agreement were sub-classified as *unanimous*. Borderline or marginal cases are defined as those photos in the testing set where the three experienced graders did not unanimously agree on presence or absence of clinical activity. Kappa statistics on TF grades and, separately, TI grades, were calculated for each of the 18 trainees on the full set of 200 photographs by comparing the trainee's grade with the consensus grade. Kappa statistics were then recalculated on the unanimous subset of cases only. Bootstrap 95% confidence intervals were determined by resampling trainees (*n* = 999).

Equation 1 [16], [17]

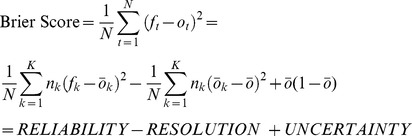
Brier score and reliability error for TF and TI were separately calculated for each trainee. (Equation 1). The Brier score can be decomposed into three component parts: reliability error, resolution, and uncertainty. Resolution and uncertainty were not analyzed in this study. Reliability error was calculated by placing the *N = 200* cases into *K = 4* mutually exclusive bins, representing forecast probabilities. Cases that the three experienced graders unanimously agreed were not TF were placed into the “0/3 TF activity” bin. Cases which one, two, or three experienced graders called as TF, were placed into the “1/3 TF activity”, “2/3 TF activity”, and “3/3 TF activity” bin, respectively. *n_k_* is the number of cases in the bin, *f_k_* is the forecast probability for that bin (either 0, 1/3, 2/3, 3/3) and 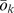 is the observed frequency of TF for the bin (ie proportion of cases in that bin the trainee graded as TF). Reliability error was sub-analyzed to reveal the proportion of cases trainees called as TF in each bin (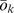 term from Equation 1); mean values across all 18 trainees for each bin are reported here with bootstrapped 95% confidence intervals (*n* = 999). Calculations were repeated for the clinical grade of TI.

To compare the effect of bin size, reliability error was recalculated for TF using *K* = 3 bins. Cases for which there was unanimous agreement amongst our 3 experienced graders as being not TF were put in the 0/2 TF activity bin. Cases for which there was unanimous agreement amongst experienced graders as TF were placed in the 2/2 TF activity bin. Cases which had any level of disagreement amongst experienced graders were placed in the 1/2 TF activity bin (equivalent to combining 1/3 TF and 2/3 TF activity bins into a single bin).

Linear regression was used to assess the relationships of these measures with each other. All calculations were performed in *Mathematica* 9.0 (Wolfram Research, Champaign, Illinois).

## Results

Out of 200 cases, the three experienced graders all agreed 76 were not TF (39% of cases) and 80 were TF (40%), giving unanimous agreement to 156 cases (79%) while disagreeing on 44 cases (21%). When assessing inter-grader agreement on the full set of 200 cases, the mean kappa score of the 18 trainees for TF was 0.774 (95% CI 0.746 to 0.800). Restricting the assessment to cases for which there was unanimous agreement amongst the 3 experienced graders (156 out of 200 cases), the mean kappa increased to 0.896 (95% CI 0.861 to 0.926). The difference in mean kappa scores was 0.122 (95% CI 0.108 to 0.136) higher when restricting analysis to the unanimous subset of cases.

With TI grading of the full set of 200 cases, the three experienced graders all agreed 98 were not TI (49% of cases) and 51 were TI (25.5%), giving unanimous agreement to 149 cases (74.5%) and disagreeing on 51 cases (25.5%). Mean kappa across 18 trainees for TI was 0.707 (95% CI 0.671 to 0.744) on the full set of 200 cases. Restricting analysis to the unanimous subset (149 out of 200 cases), the trainees' mean kappa for TI increased to 0.795 (95% CI 0.756 to 0.833). The difference in mean kappa scores was 0.088 (95% CI 0.070 to 0.107) higher for the unanimous subset.

Reliability error and Brier score for TF were calculated for the 18 trainees using the 200 cases placed into 4 bins. The three experienced graders unanimously agreed 78 cases were not TF (0/3 TF activity bin) and 80 cases were TF (3/3 TF activity bin). There were 18 cases which were called TF by only 1 experienced grader (1/3 TF activity bin) and 26 cases which were called TF by 2 experienced graders (2/3 TF activity bin). Mean reliability error for the 18 trainees on the full set of cases was 0.013 (95% CI 0.007 to 0.021). Mean reliability error for the unanimous subset (i.e. the 156 cases in the 0/3 TF activity and 3/3 TF activity bins) was 0.009 (95% CI 0.004 to 0.018). The difference in mean reliability error was 0.004 (95% CI 0.001 to 0.006) higher for the full set as compared to the unanimous subset. Mean Brier score for TF on the full set of cases was 0.089 (95% CI 0.078 to 0.101). Mean Brier Score the unanimous subset (i.e. 2 bins) was 0.052 (95% CI 0.038 to 0.069). The mean Brier score was 0.037 (95% CI 0.033 to 0.040) higher on the full set of cases for TF grading.

Reliability error and Brier score for TI were calculated for the 18 trainees using the full 200 cases placed into 4 bins. The three experienced graders unanimously agreed 98 cases were not TI (0/3 TI bin) and 51 cases were TI (3/3 TI bin). There were 40 cases which were called TI by only 1 experienced grader (1/3 TI bin) and 11 cases which were called TI by 2 experienced graders (2/3 TI bin). Mean reliability error for TI on the full set of cases was 0.034 (95% CI 0.025 to 0.045). Mean reliability error on just the unanimous subset (i.e. 2 bins) was 0.025 (95% CI 0.018 to 0.035). The difference in mean reliability error was 0.009 (95% CI 0.005 0.013) higher for the full set. Mean Brier score on the full set of cases was 0.110 (95% CI 0.098 to 0.122). Mean Brier Score for the unanimous subset (i.e. the 0/3 and 3/3 bins) was 0.087 (95% CI 0.070 to 0.104). The mean difference in Brier score was 0.023 (95% CI 0.019 to 0.029) higher on the full set of cases for TI.

The mean proportion of cases the 18 trainees scored as TF in the 0/3 TF activity bin, which contained the 76 cases that all three experienced graders scored as normal, was 6.9% (95% CI 3.9% to 10.5%). Mean proportion of cases called TF in the 1/3 TF activity bin, containing the 18 cases which only 1 experienced grader called TF, was 50.9% (95% CI 43.8% to 57.7%). Mean proportion of cases scored as TF in the 2/3 TF activity bin, containing the 26 cases that 2 experienced graders called TF, was 80.1% (95% CI 75.0% to 85.7%). Mean proportion of cases called TF in the 3/3 bin, containing the 80 cases all experienced graders called TF, was 96.5% (95% CI 95.7% to 97.4%).

Similarly for TI, the mean proportion of cases the 18 trainees scored as TI in the 0/3 TI bin, which contained the 98 cases that all three experienced graders scored as normal, was 0.6% (95% CI 0.06% to 1.24%). Mean proportion of cases called TI in the 1/3 TI bin, containing the 40 cases which only 1 experienced grader called TI, was 11.8% (95% CI 6.3% to 17.8%). Mean proportion of cases scored as TI in the 2/3 TI bin, containing the 11 cases that 2 experienced graders called TI, was 46.5% (95% CI 37.4% to 55.1%). Mean proportion of cases called TI in the 3/3 bin, containing the 51 cases all experienced graders called TI, 75.8% (95% CI 71.5% to 80.3%).

As an example, we report kappa scores and reliability errors for TF for 2 individual graders. Grader A had a kappa of 0.736 and a reliability error of 0.033. %TF in the 0/3 TF, 1/3 TF, 2/3 TF and 3/3 TF activity bins were 14.5%, 72.2%, 96.1%, and 98.8%, respectively. Grader B had a kappa of 0.739 and a reliability error of 0.005. %TF in the 0/3 TF, 1/3 TF, 2/3 TF and 3/3 TF activity bins were 5.3%, 50%, 57.7%, and 97.5% respectively. Thus trainees with similar kappas may have different reliability scores.

Reliability error for TF grades was recalculated by categorizing the 200 cases into 3 bins, instead of 4, by merging the 1/3 and 2/3 TF activity bins into a single 1/2 TF activity bin. The three experienced graders unanimously agreed 76 cases were not TF (0/2 TF bin) and 80 cases were TF (2/2 TF bin). There were 44 cases which the experienced graders disagreed on (1/2 TF bin). Mean reliability error for TF on the full set of cases in 3 bins was 0.008 (95% CI 0.004 to 0.014). Mean reliability error on just the unanimous subset (i.e. 2 bins) was 0.009 (95% CI 0.004 to 0.018). The difference in mean reliability error across all 18 trainees between the full set of cases (all 3 bins) and the unanimous subset (2 bins: 0/2 and 2/2 TF activity) was 0.001 (95% CI −0.002 to 0.004). The mean proportion of cases the 18 trainees scored as TF in the 0/2 TF activity bin, which contained 76 cases that all three experienced graders scored as normal, was 6.9% (95% CI 3.9% to 10.5%). The mean proportion of cases called TF in the 1/2 TF activity bin, containing 44 cases which the three experienced graders disagreed on, was 68.2% (95% CI 62.9% to 73.1%). Mean proportion of cases called TF in the 2/2 bin, containing 80 cases all experienced graders called TF, was 96.5% (95% CI 95.7% to 97.4%).

**Figure 1:**
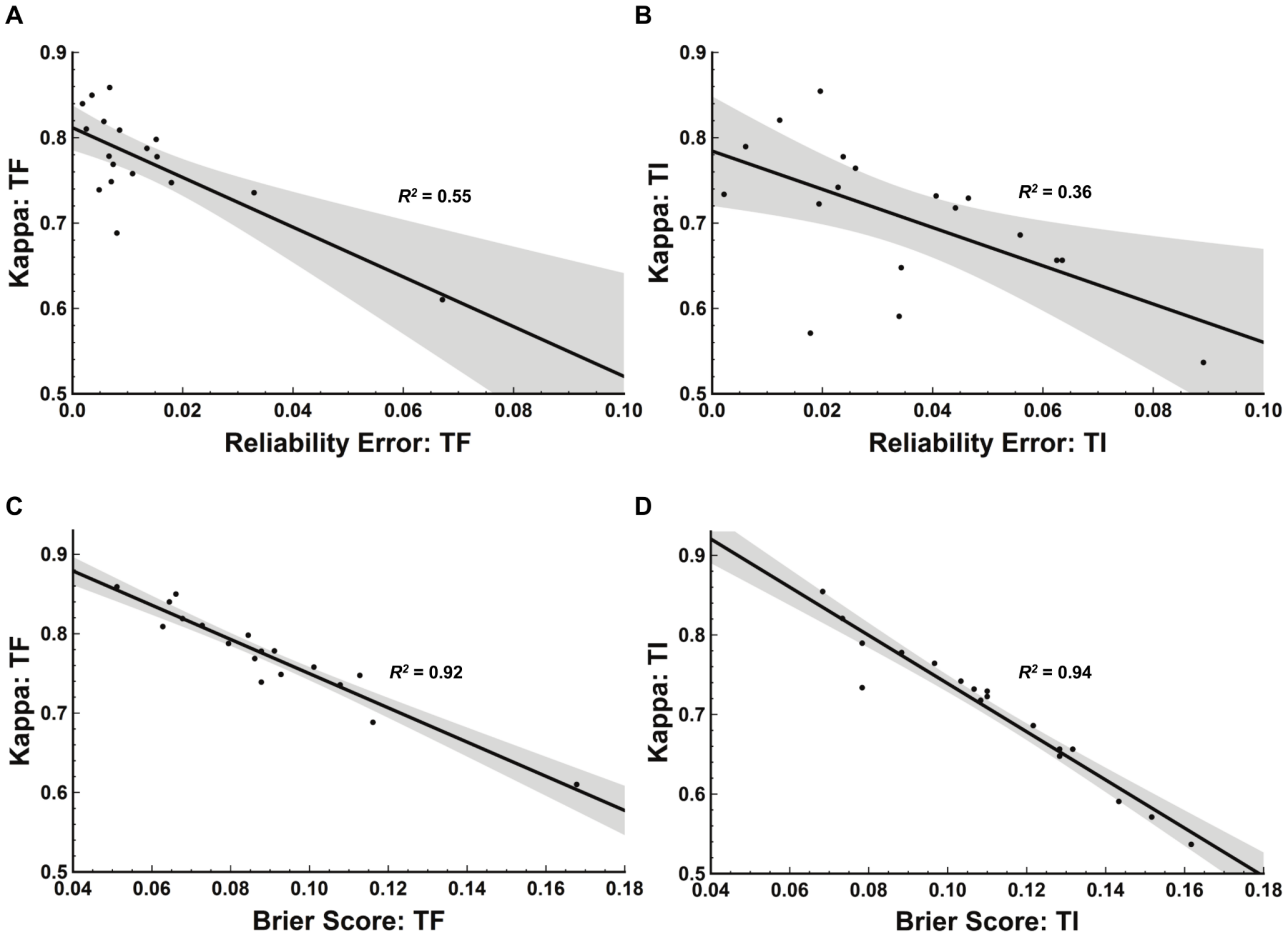
Relationships between measures of inter-grader agreement. This figure shows the relationships between kappa and reliability error for trachomatous inflammation – follicular (TF) grades (A), kappa and reliability error for trachomatous inflammation – intense (TI) grades (B), kappa and Brier score for TF (C), and kappa and Brier score for TI (D). Scatter plot points indicate results for each of the 18 trainees using the full set of 200 cases. Solid black line indicates linear regression fit. Grey shading indicates the estimated 95% confidence interval bands.

Figure 1 depicts the relationships between our calculated measures on the full set of 200 cases. Figure 1A and 1B show a loose correlation between reliability error and kappa for TF (*R^2^* = 0.55) and a weak correlation for TI (*R^2^* = 0.36). Brier score and kappa are much more highly correlated, as Figure 1C and 1D show with *R^2^* = 0.92 for TF and *R^2^* = 0.94 for TI.

## Discussion

As shown elsewhere with a different test set and trainees [19], we found higher agreement with a kappa statistic when analysis was limited to those cases with unanimous agreement amongst experienced graders. Removing the cases where experienced graders disagreed led to a 15.7% increase in mean kappa scores for the 18 trainees for TF grades and 12.4% increase for TI grades. Kappa has been the traditional method for assessing inter-grader agreement and for certifying trachoma field graders [13], [14], [20]. Proper training sets should contain the full spectrum of disease, presumably matching field conditions, not just easy-to-grade cases. Otherwise agreement cannot be expected to be as high as found during training and testing.

When the kappa statistic is used to compare a trainee's score with a gold-standard, it is essentially a scaled accuracy, with the relationship between kappa and accuracy perfectly linear when the prevalence of disease is 50%, and close to linear otherwise. Kappa requires marginal cases be classified as either having clinical activity or not. In contrast, reliability error treats cases as having a probability of possessing activity, which here we set equal to the proportion of 3 experienced graders scoring that case active. Reliability error assesses how close the proportion of positive observed outcomes, given a forecast probability, are to that forecast probability. For a trainee to have perfect (ie 0) reliability error on the 200 cases in this study, he/she must grade no cases in the 0/3 TF activity bin as TF (0 out of 76 cases), one-third in the 1/3 TF activity bin (6 out of 18 cases), two-thirds in the 2/3 TF activity bin (approximately 17 out of 26 cases), and all in the 3/3 TF activity bin (80 out of 80 cases). In contrast to kappa and accuracy, reliability error does not assess the individual grades a trainee gives for borderline cases in the 1/3 and 2/3 TF activity bins; rather, it assesses the proportion of cases scored active in those bins overall.

Brier score is highly correlated to kappa (Figure 1 C,D), and thus provides little or no additional information. However reliability error, derived as a portion of the Brier score, does capture information not found in kappa—as evidenced by our finding that reliability error and kappa are not well-correlated (*R^2^* = 0.56 for TF and *R^2^* = 0.36 for TI).

In contrast to kappa, reliability error can be constructive. We expect the proportion of TF or TI called in the 0/3, 1/3, 2/3 and 3/3 activity bins to be 0%, ∼33%, ∼66%, and 100% respectively. We found for TF, mean activity (across all 18 trainees) in those bins to be 6.9%, 50.9%, 80.1%, and 96.5%, respectively. For TI, the mean activity was 0.6%, 11.8%, 46.5%, and 75.8%. Thus, there was a tendency for our trainees to over-call TF and under-call TI, especially marginal cases (1/3 and 2/3 activity TF and TI bins). The proportion of activity called in each bin could be used, at the level of the trainee, to specifically refine scoring over portions of the disease spectrum, making reliability error a constructive measure. For example, two graders had nearly identical kappas (0.736 and 0.739), but reliability errors nearly 7-fold different (0.033 vs 0.005). One grader clearly over-called obviously normal cases (0/3 TF bin) as well as moderate cases of TF (1/3 and 2/3 TF bin). We used this information to remediate the grader's tendency to over-call clinical activity.

Furthermore, in contrast to kappa scores, reliability error scores are not necessarily subject to reduction by inclusion of borderline cases. Though we see a statistically significant difference in mean 4-binned reliability error between the full set of cases and the unanimous subset for both TF and TI, our trainees disproportionately over-called TF and under-called TI grades for borderline cases (the 1/3 and 2/3 activity bins). In our re-calculation with 3 bins for TF, there was no statistically significant difference in mean reliability error scores between the full set of cases and the unanimous subset. Though trainees tended to over-call TF in this recalculation, the borderline cases (1/2 TF activity bin) were not disproportionately over-called. Further studies must be done to determine an optimal number of bins to use when calculating reliability error for trachoma grades.

Our study has limitations which may affect generalizability. We only analyzed cases from a specific hyper-endemic region in Niger. Other countries may have a different spectrum of disease. We had 3 experienced graders score the 200 cases; there may be variability among other experienced graders on these 200 cases. Additionally, using a larger number of experienced graders may allow for better resolution in categorizing cases as marginal. We used 4 bins to categorize cases, based on the proportion of the three graders that scored the case as having activity. A different binning procedure can demonstrate different results, as discussed previously in the 3-bin recalculation. Conjunctival photographs were used to train graders and perform this study. Field examination has several advantages over photo grading, including that the conjunctiva may be examined from multiple angles, is always in focus, and illumination can be adjusted. The conjunctiva is a three-dimensional structure, particularly when inflamed, whereas a photograph is a two-dimensional representation [15]. For the purposes of our study, however, testing 18 graders in the field on the same cases would not have been feasible. Lastly, this study looked at reliability of trachoma grading using the WHO's simplified system currently used by most trachoma programs. We may have seen different results using the expanded classification system [12].

Because of its relatively low cost, trachoma control programs will likely continue using clinical examination to make treatment decisions. Thus proper training of field graders is important. To ensure high-quality grading, these graders should be trained on the full spectrum of disease that they are likely to encounter in the field. Using the kappa statistic to judge certification can be difficult to interpret, depending on how widely experienced graders disagree on cases in the test set, given that inclusion of marginal cases tends to deflate apparent agreement. If even experts disagree, a trainee's answer may reveal little and lower their inter-grader agreement, as assessed by a kappa statistic. However, information can be learned about how a grader is assessing marginal cases by looking at the breakdown of their reliability error. Further studies can help determine if reliability error would also be an important metric to certify graders.
